# Integration of single-cell and bulk analysis reveals TBXAS1 as a key platelet-related gene causing poor prognosis in osteosarcoma

**DOI:** 10.3389/fgene.2024.1519529

**Published:** 2024-12-09

**Authors:** Han Liu, Wacili Da, Jianhua Mu, Xuanhong He, Zhuangzhuang Li, Taojun Gong, Jingjing Wang, Li Min, Minxun Lu, Chongqi Tu

**Affiliations:** ^1^ Department of Orthopedics, Orthopaedic Research Institute, West China Hospital, Sichuan University, Chengdu, China; ^2^ Department of Orthopedics Surgery, Orthopeadic Research Institute, West China Hospital, West China Medical School, Sichuan University, Chengdu, Sichuan, China; ^3^ Department of Endocrine, Sichuan Provincial People’s Hospital, School of Medicine, University of Electronic Science and Technology of China, Chengdu, China

**Keywords:** osteosarcoma, blood platelets, single cell, macrophages, prognosis

## Abstract

**Background:**

Platelets are associated with poor prognosis in most tumors, but their specific pathogenic mechanism in osteosarcoma is not yet clear. The objective of this study is to conduct an in-depth analysis of how genes closely related to platelet function impact the prognosis of osteosarcoma patients. We hope that through this research, we can uncover the potential mechanisms of these genes in the development and progression of osteosarcoma, thereby providing new therapeutic strategies and theoretical foundations for improving the prognosis of osteosarcoma patients.

**Method:**

We collected the blood routine test data of patients who were initially diagnosed with osteosarcoma at the Department of Bone Tumors, West China Hospital, from January 2012 to January 2022. By applying the LASSO-COX regression analysis, a statistical method, we found that the platelet count is associated with the prognosis of osteosarcoma patients. To further explore this relationship, we obtained single-cell data and bulk RNA data of osteosarcoma patients from the TARGET database and GEO database, respectively. By analyzing these data, we revealed at the transcriptomic level how platelets contribute to the poor prognosis in osteosarcoma patients.

**Result:**

Platelets are associated with the prognosis of osteosarcoma patients (HR = 3.9, 95% CI = 1.9–8.1, *P* < 0.001). Through the analysis of transcriptomic data from the TARGET database and GEO database, we found significant heterogeneity in tumor-specific pathways and immune infiltration under different platelet-related gene expression patterns. Among these, TBXAS1 was identified as a key gene that affects the prognosis of osteosarcoma patients. In addition, single-cell data analysis showed that the platelet-related gene TBXAS1 is mainly enriched in macrophages, and markers of macrophages are significantly associated with poor prognosis in osteosarcoma patients.

**Conclusion:**

TBXAS1 is a key platelet-related gene that leads to poor prognosis in osteosarcoma, and this gene may affect the prognosis of osteosarcoma patients by interacting with macrophages.

## Introduction

Osteosarcoma is the most common primary bone tumor in children, adults, and elderly people over 60 years old (Damron et al., 2007; Kumar et al., 2018). Characterized by poor prognosis and high disability rates, the adverse prognosis of osteosarcoma is mainly due to its high tendency to metastasize, especially to the lungs (Huang et al., 2019; Sajadi et al., 2004). In the general population, the incidence of osteosarcoma is 2–3 cases per million per year, with the disease occurring in individuals aged 0–14 years and over 60 years. Males have a 1.4 times higher likelihood of developing the disease than females (Kumar et al., 2018). The etiology of osteosarcoma remains unknown in most people. Follow-up data show that the 5-year survival rate for osteosarcoma patients without lung metastasis is 70%, while the 5-year survival rate for patients with lung metastasis drops to 30% (Kaste et al., 1999). Despite advances in the treatment of osteosarcoma, such as chemotherapy, neoadjuvant chemotherapy, radiotherapy, and immunotherapy, the 5-year survival rate for osteosarcoma patients is still far from satisfactory. The pathophysiology of osteosarcoma has not been clearly elucidated, but many studies have shown that its pathogenesis is closely related to genetic factors. Currently, most main treatment methods require drug chemotherapy in addition to surgery. Based on the need to elucidate the pathogenesis and find more effective chemotherapeutic drugs, the screening and identification of osteosarcoma biomarkers has become a research hotspot.

Platelets, which regulate hemostasis and thrombosis, are one of the three main types of blood cells in the human body (Pavlovic et al., 2019). Platelets are produced in the bone marrow and circulate in the blood. When bleeding or injury occurs, they quickly gather at the wound site to form a thrombus and recruit more platelets. Platelets not only play an indispensable role in coagulation and maintaining hemostasis but are also directly related to cancer. It is well known that platelets are significantly involved in the growth and metastasis of cancer. In 1872, Leopold Riess discovered that thrombocytosis is commonly found in solid tumors (Pardo et al., 2017). In many cancer patients, the platelet count significantly increases (Haemmerle et al., 2018). As a key component of the osteosarcoma microenvironment, platelets participate in the growth and metastasis of osteosarcoma, and therefore have great potential in targeted therapy for osteosarcoma (Haemmerle et al., 2018). Recent studies have shown that platelets play a central role in the systemic and local responses to tumor growth (Haemmerle et al., 2018). Platelets are capable of protecting tumor cells, making them resistant to attacks by immunotherapy (He et al., 2017). As platelets flow through the circulatory system, they assist tumor cells in adhering to the endothelium when these cells become trapped at the site of metastasis, thereby facilitating the occurrence of metastasis (Pavlovic et al., 2019). The unique biological characteristics of platelets enable them to participate in the immune response to tumor cells (Jochems et al., 2017). More than 30% of patients with malignant solid tumors also exhibit concomitant thrombocytosis, which reduces their survival rate (Zhou et al., 2020). This phenomenon occurs when tumor-derived IL-6 stimulates an increase in thrombopoietin levels, leading to thrombocytosis (Stone et al., 2012). Increased platelet activity promotes tumor growth and metastasis and reduces the effectiveness of immunotherapy against tumor cells (He et al., 2017). Platelets affect the therapeutic outcomes for patients and are involved in multiple steps of cancer metastasis. Additionally, platelets can prevent chemotherapy-induced apoptosis of cancer cells. It has been proven that thrombocytosis is closely associated with poor responses to chemotherapy during both *in vitro* and *in vivo* experiments, suggesting that if we inhibit the number or activity of platelets, we can enhance the efficacy of chemotherapeutic drugs (Bottsford-Miller et al., 2015). However, some studies have shown that platelets also have an anti-tumor effect. Platelets inhibit tumor growth by transporting mir-24 to cancer cells targeting mt-Nd2 and Snora75, indicating that the regulatory effect of platelets on tumor growth is diverse. Therefore, the specific role of platelets in osteosarcoma needs further study (Michael et al., 2017).

In this article, we used LASSO-COX regression analysis to identify that platelets contribute to poor prognosis in patients with osteosarcoma, and we validated this finding using transcriptomic data from public databases. Additionally, we found that the platelet-related gene TBXAS1 is a key pathogenic gene in osteosarcoma, and its interaction with macrophages may be one of the reasons why platelet-related genes promote the progression of osteosarcoma.

## Materials and method

### Patients

With the approval of the Medical Ethics Committee, we reviewed the clinical data of osteosarcoma patients from January 2012 to January 2022 in the database of the Musculoskeletal Tumor Center of West China Hospital. During the review process, we included and excluded patients according to the following criteria: 1) patients with high grade osteosarcoma confirmed by histopathology; 2) patients have complete hematological test results before neoadjuvant chemotherapy; 3) patient received standard treatment at West China Hospital. The exclusion criteria: 1) Patients with histopathologically confirmed low-grade osteosarcoma (intramedullary and bone surface) and periosteal osteosarcoma; 2) Patients who had received neoadjuvant chemotherapy before their first-time consultancy in our hospital; 3) patients with hematological diseases; 4) patients with other malignancies; 5) patients not received standard treatment (patients who are misdiagnosed and mistreated or fail to complete postoperative chemotherapy). Finally, 150 patients were included in our study after passing the inclusion and exclusion criteria. Each patient was followed up regularly until death or January 2022. The following follow-up principles were followed: reexamination every 3 months within 1 year after surgery; reexamination every 4 months 1–2 years after surgery; reexamination every 5 months 2–3 years after surgery; reexamination every 6 months 3–5 years after surgery; reexamination every year more than 5 years after surgery.

### Data collection

Firstly, univariate cox regression analysis was used to screen indicators of prognostic value in the overall cohort. Then, Single-cell datasets of osteosarcoma with the numbers GSE143753, GSE152048, and GSE162454 were obtained from the Gene Expression Omnibus (GEO) database (https://www.ncbi.nlm.nih.gov/geo/). Cells were filtered using the R package Seurat with the following criteria: cells with fewer than 200 or more than 7,500 expressed genes were excluded, and cells with more than 10% of UMI mapping to mitochondrial genes were also excluded. Only genes expressed in at least five cells were retained. Subsequently, the data was normalized and the top 2,000 highly variable genes were detected using the “FindVariableFeatures” function (Ljunggren and Johansson, 1988).

Next, based on the 2,000 genes, the dimensionality of the scRNA-seq data was reduced by PCA, and 40 principal components were selected for subsequent analysis using the UMAP algorithm. Finally, cell clustering was performed using the “FindClusters” function. To determine cell types, cells were annotated according to the CellMarker database and the corresponding literature (Hu et al., 2023). In addition, bulk RNA data of osteosarcoma with the number GDC TARGET-OS was obtained from the UCSC Xena database (https://xena.ucsc.edu). Platelet-related genes were collected and compiled from the MSigDB database (http://www.broad.mit.edu/gsea/msigdb/) and included genes involved in platelet activation, aggregation, and signaling transduction.

### Differential analysis

DESeq2 is a method for differential analysis of count data that uses shrinkage estimation of dispersions and fold changes to improve the stability and interpretability of the estimates. This allows for a more quantitative analysis, focusing on the intensity of differential expression, rather than just the presence of differential expression (Love et al., 2014). We used the DESeq2 package to perform differential analysis on the counts data of osteosarcoma. For single-cell data, the FindMarkers function was used to identify DEGs (adjusted *P*-value <0.05 and fold change [FC] >2) between two clusters.

### Enrichment analysis

Enrichment analysis was conducted to determine whether a series of pre-defined biological processes were enriched. Gene Ontology (GO) analysis was performed using the R package clusterProfiler (version 4.2.2), which is a general tool for interpreting omics data. To ensure enough genes for Gene Ontology (GO) analysis (Ashburner et al., 2000), we relaxed the criteria for expression scores compared to the differential expression analysis. Genes expressed in at least 25% of cells in a cluster were used for GO analysis. Only the top five enriched biological process items for each cell type were shown in the analysis. Pathways with adjusted *P*-values less than 0.05 were considered significant.

### Cell communication analysis

To visualize and analyze the intercellular communication in our data, we performed a CellChat analysis using the “CellChat” package (Jin et al., 2021), which is notable for its ability to integrate intercellular ligand-receptor communication with intracellular transcription factor expression, forming a ligand-receptor-transcription factor axis (L-R-TF axis). It also includes pathway activity analysis, allowing the analysis of receptor-cell pathway changes caused by the communication between two specific cell types. We created a new CellChat object from the Seurat object. Cell types had been added to the CellChat object as cell metadata. CellChat identified differentially overexpressed ligands and receptors for each cell group and associated each interaction with a probability value to quantify the communication between two cell groups mediated by these signaling genes. Significant interactions were identified based on a statistical test that randomly permutes the group labels of cells and then recalculates the interaction probabilities. The results were visualized using netVisual_bubble. Additionally, the cell communication results for different prognostic scores were summarized and compared. The analysis of cell communication in spatial transcriptomics data also used the “CellChat” package.

### Machine learning methods were used to predict prognosis

To explore the predictive effect of specified genes on patient prognosis, we constructed a prognostic model using the expression matrix of the specified genes. First, we integrated 10 classic algorithms: Random Forest (RSF), Least Absolute Shrinkage and Selection Operator (LASSO), Gradient Boosting Machine (GBM), Survival Support Vector Machine (Survival-SVM), Supervised Principal Components (SuperPC), Ridge Regression, Cox Partial Least Squares Regression (plsRcox), CoxBoost, Stepwise Cox, and Elastic Net (Enet). Among them, RSF, LASSO, CoxBoost, and Stepwise Cox have dimensionality reduction and variable selection functions, and we combined them with other algorithms to form 87 machine learning algorithm combinations. Next, we used random sampling to extract 421 samples (70%) as the training cohort and used these 47 combinations to construct signatures in an expression file with 54 platelet-related genes. Finally, we calculated the C-index for each cohort using the features obtained in the training cohort. Based on the average C-index of the training and testing cohorts, we ultimately selected the optimal prognostic model for predicting clear cell renal cell carcinoma (ccRCC) using platelet-related genes.

### Gene SET score

We used the AddModuleScore function to calculate specific scores for individual cells. In addition, we performed a GSVA evaluation using the MSigDB database (http://www.gsea-msigdb.org/) to assess the enrichment of characteristic pathways in each cell type (Liberzon et al., 2011). The TIDE score was calculated using the Tumor Immune Dysfunction and Exclusion (TIDE, http://tide.dfci.harvard.edu/login/) database (Valera et al., 1993; Chen Y. et al., 2021).

### Statistical analysis

In our study, bioinformatics analysis and visualization were performed using R software (version 4.1.2). Pearson correlation and Spearman correlation were used for correlation analysis. The specific methods are described in the results section and in the legends of the figures. Statistical significance was defined as *P < 0.05, **P < 0.01, ***P < 0.001, and *P* < 0.05 was considered statistically significant. For multiple hypothesis testing, the Benjamini-Hochberg method was used to adjust *P* values.

## Result

### Platelet-related genes are highly expressed in tumor tissues, especially in high-grade osteosarcoma

**FIGURE 1:**
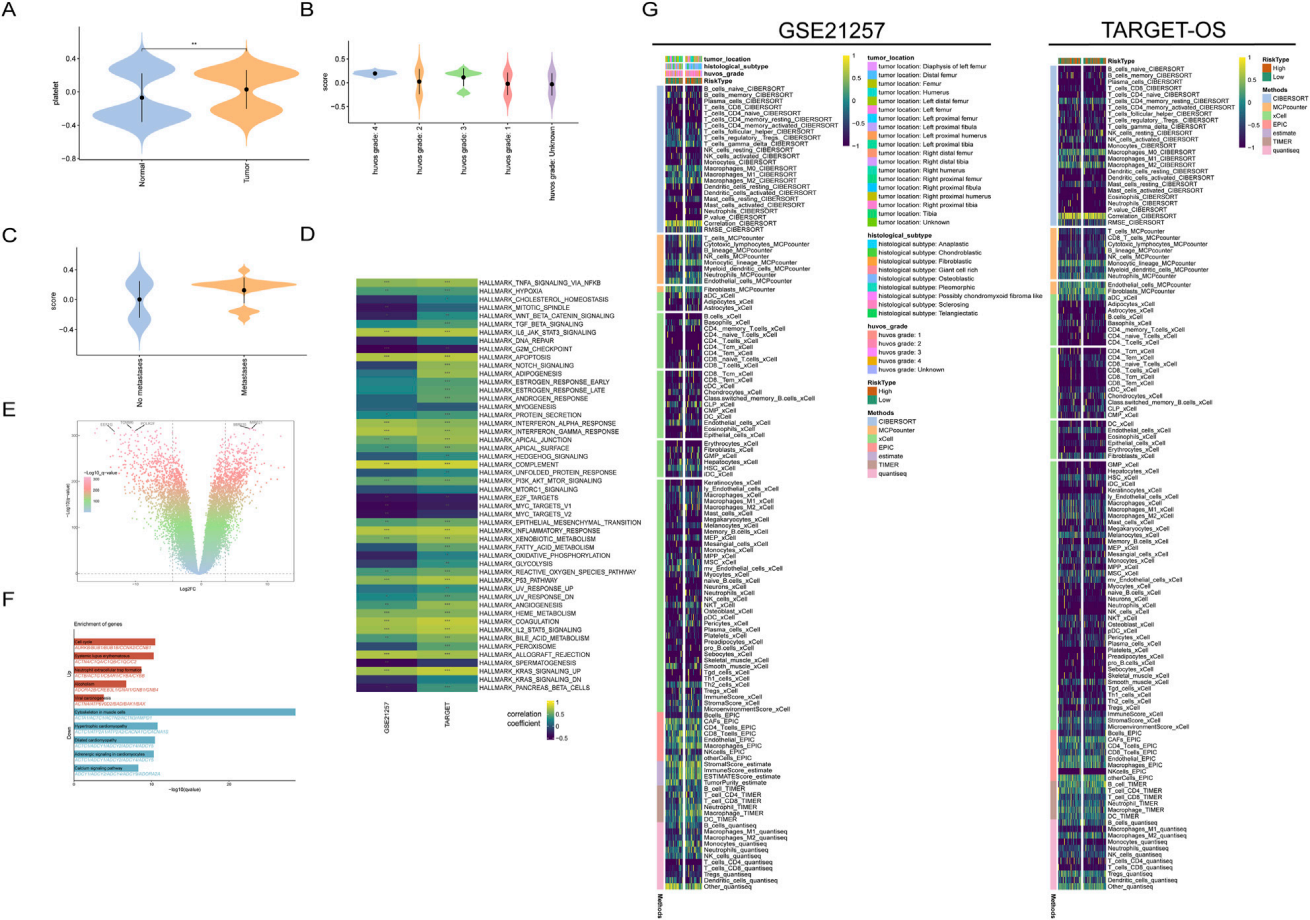
Transcriptional characteristics of platelet-related genes in osteosarcoma. **(A)** Platelet-related gene scores in normal and tumor tissues; **(B)** Platelet scores in osteosarcoma patients with different HuVos grades; **(C)** Patients with metastatic osteosarcoma have higher platelet scores; **(D)** HALLMARKER scores of patients in two datasets; **(E)** Differential analysis volcano plot integrating osteosarcoma data from the TARGET-OS dataset and normal muscle tissue data from the GTEx dataset; **(F)** Enrichment analysis of tumor samples relative to normal tissue; **(G)** Immunological infiltration scores and relative clinical information.

Many studies have shown that platelets have an adverse effect on the prognosis of patients in most tumors. This is consistent with the results of our analysis. To explore the tissue-specific characteristics of platelet-related genes in osteosarcoma, we screened platelet-related genes with scores exceeding 20 from the GeneCards database. After preprocessing the data from public databases (Supplementary Figure S1A), we used the GSVA (Gene Set Variation Analysis) package to score the expression of platelet-related genes for each patient. We compared the platelet scores between normal and tumor tissues in patients, and the results showed that the platelet score in tumor tissues was significantly higher than in normal tissues (Figure 1A, *P* < 0.01).

This finding confirms to some extent the enrichment of platelet-related genes in the tumor tissues of osteosarcoma, and these genes may have a synergistic effect on tumor development. Interestingly, the platelet scores in both tumor and normal tissues showed a bimodal distribution, indicating that the transcriptional characteristics of platelet-related genes may have two different modes. In the GSE21257 dataset, the higher the Huvo’s grade of tumor patients (Figure 1B), the higher the platelet-related genes. In addition, patients with metastatic osteosarcoma had higher platelet scores (Figure 1C). These results suggest that platelet scores are closely related to tumor behavior in osteosarcoma patients.

Therefore, to explore the specific mechanisms by which platelet-related genes play a role in osteosarcoma, it is necessary to analyze related signaling pathways. The HALLMARK50 gene set was obtained from the MsigDB database, which is a collection of 50 gene sets that can evaluate the activity of various important signaling pathways in tumors. Using this gene set for scoring can represent the activation status of the corresponding pathway to some extent. After scoring the tumor patients in the TARGET-OS dataset and the GSE21257 dataset separately, we conducted a correlation analysis between the HALLMARK50 scores and the corresponding platelet scores for each patient. The results showed that the correlation coefficients of the two datasets were highly consistent, indicating that there are certain commonalities among different osteosarcoma patient populations, in addition to heterogeneity (Figure 1D).

In both datasets, the scores of the epithelial-mesenchymal transition pathway were significantly positively correlated with the platelet scores. The epithelial-mesenchymal transition is a depolarization process that plays an important role in tumor metastasis, which may also be the reason for the higher platelet scores in patients with metastatic osteosarcoma.

To further screen for platelet-related genes that are significantly differentially expressed in tumor tissues, we integrated the osteosarcoma data from the TARGET-OS dataset and the normal muscle tissue data from the GTEx dataset. After removing batch effects, we used the “limma” package for differential analysis, selecting genes with *P* < 0.05 and |LogFC|>2. This resulted in a total of differentially upregulated genes and differentially downregulated genes. Enrichment analysis of these differentially expressed genes revealed that the upregulated genes were mainly enriched in the cell cycle, which is consistent with the characteristic of abnormal proliferation in tumors (Figures 1E, F).

Tumor immune escape is one of the important conditions for tumor occurrence. To further understand the relationship between platelet score and the tumor immune microenvironment, we used various immune infiltration calculation methods to assess the immune cell infiltration scores in osteosarcoma patients with different platelet scores. In the TARGET-OS dataset and GSE21257 dataset, osteosarcoma patients were divided into high platelet activity and low platelet activity groups based on the median platelet score.

The results of immune infiltration analysis showed that there was significant heterogeneity in the infiltration of immune cells in osteosarcoma between the two datasets. Additionally, the infiltration score of CD8^+^ T cells, which play a crucial role in anti-tumor immunity, was significantly lower in osteosarcoma tissues compared to other immune cells. This suggests that there may be abnormal immune crosstalk, which could be one of the reasons for the poor prognosis of osteosarcoma patients (Figure 1G). Furthermore, the high macrophage score analyzed by the XCELL method was associated with poor prognosis in osteosarcoma patients (Supplementary Figure S1B).

### Platelet-related genes can predict the prognosis of patients

**FIGURE 2:**
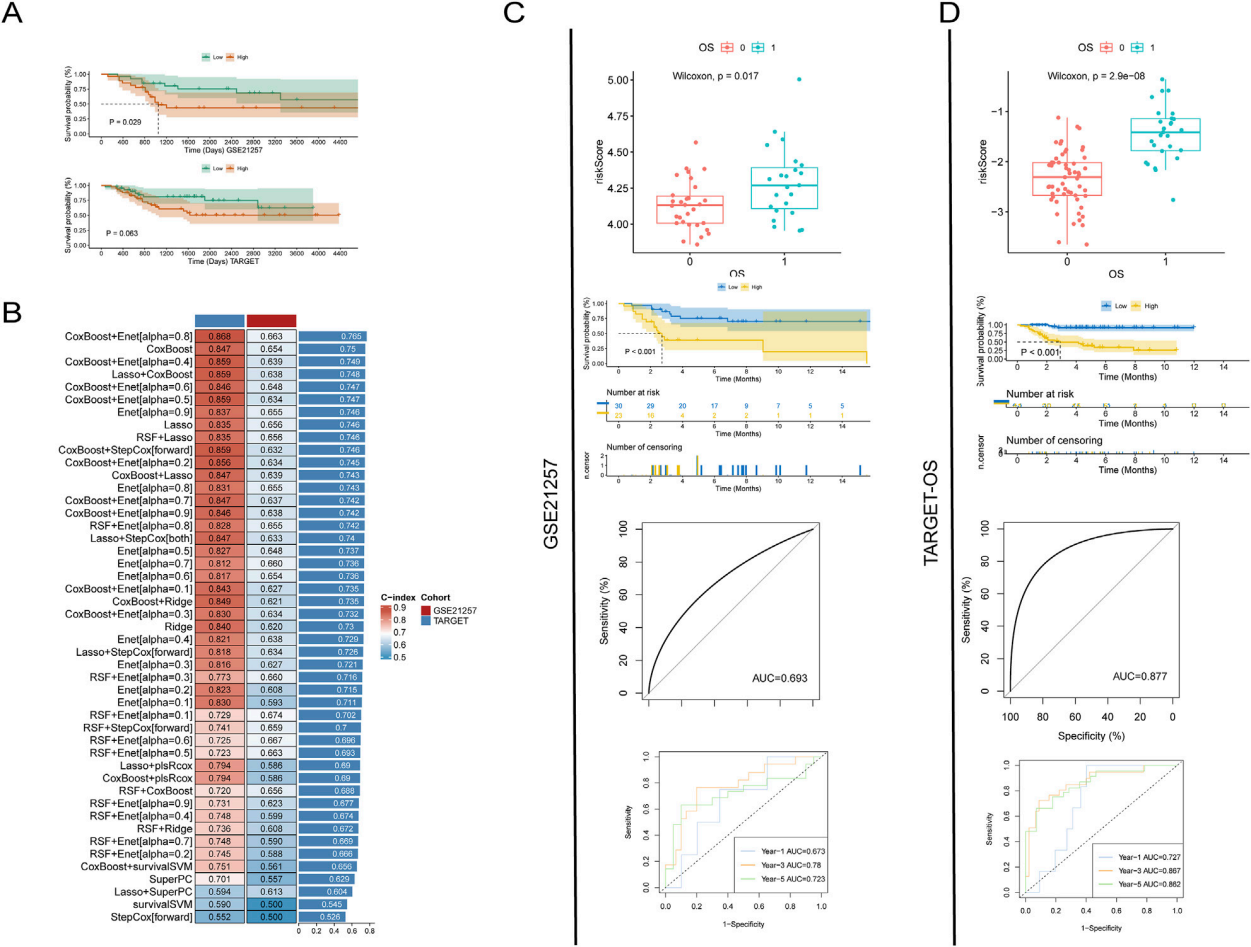
Prognostic analysis of platelet-related genes. **(A)** Survival analysis after grouping by platelet score; **(B)** Evaluation of the prognostic predictive power of platelet genes using various machine learning methods; **(C, D)** Accuracy of predicting prognosis using LASSO analysis of platelet-related genes in two datasets. From top to bottom: risk scores for different prognostic outcomes, survival analysis for different risk scores, overall predictive power, and predictive power for 1 year, 3 years, and 5 years.

Patient prognosis is an important indicator for evaluating the effectiveness of cancer treatment, and predicting patient prognosis can also help in planning a more comprehensive and personalized treatment strategy for patients. By intersecting platelet-related genes with differentially expressed genes, we obtained differentially expressed platelet-related genes. Using GSVA, we scored the TARGET-OS dataset and GSE21257 dataset with these differentially expressed platelet-related genes and used the median score to differentiate between high and low-risk groups. The results showed that in both the TARGET-OS dataset and GSE21257 dataset, a higher platelet score was associated with a poorer prognosis, with consistent conclusions obtained from both datasets (Figure 2A). This result, along with the previous findings on the relationship between platelet score and clinical phenotype and tumor signaling pathways, further highlights the adverse promotional role of platelet-related genes in osteosarcoma patients.

In order to more accurately evaluate the efficacy of differentially expressed platelet-related genes in predicting the prognosis of patients, we used machine learning methods to train and validate the model. By combining different machine learning techniques, we can make full use of the advantages of various methods, which helps to prevent the problem of model overfitting or underfitting to a certain extent, thereby improving the prediction accuracy of the model. The prediction results indicate that the combined model of CoxBoost and Enet [alpha = 0.8] has the best predictive performance, with a C-index of 0.868 in the training set and 0.663 in the test set, resulting in an average C-index of 0.765.

However, this combination of machine learning methods can only assess the overall predictive efficacy of differentially expressed platelet-related genes and cannot be pinpointed to individual genes (Figure 2B). Therefore, we used the LASSO method to screen for genes most closely related to prognosis and calculated the impact weight of each gene on prognosis. The results showed that the risk score of dead patients calculated by LASSO method was significantly higher than that of survival patients in TARGET-OS dataset and GSE21257 dataset. The Kaplan-Meier (K-M) survival curve also showed that the prognosis of patients with low risk score was significantly better than that of patients with high risk score. The consistent results in both datasets reconfirm the trend of poorer patient outcomes with higher platelet scores.

In addition, the receiver operating characteristic (ROC) curve analysis showed that the area under the curve (AUC) value of the GSE21257 dataset was 0.693, while the AUC value of the TARGET-OS dataset was 0.877. In the 1 -, 3 -, and 5-year follow-up, the AUC values of GSE21257 dataset were 0.673, 0.78, and 0.723, respectively, while those of TARGET-OS dataset were 0.727, 0.867, and 0.862, respectively. These data show that platelet-associated transcriptomic signatures can effectively predict prognosis and perform well (Figures 2C, D).

### Higher platelet activity promotes aberrant cell communication in tumor tissues

Studying the interactions and developmental processes among different cells at the single-cell level has greatly enhanced our understanding of diseases. After collecting single-cell data from three GEO databases, we preprocessed the data to eliminate batch effects and remove cells of low quality (Supplementary Figure S1C). By performing dimensionality reduction on the cells, we were able to identify genes that were differentially upregulated in each cell (Supplementary Figure S1D) and annotate these genes based on relevant databases and literature.

**FIGURE 3:**
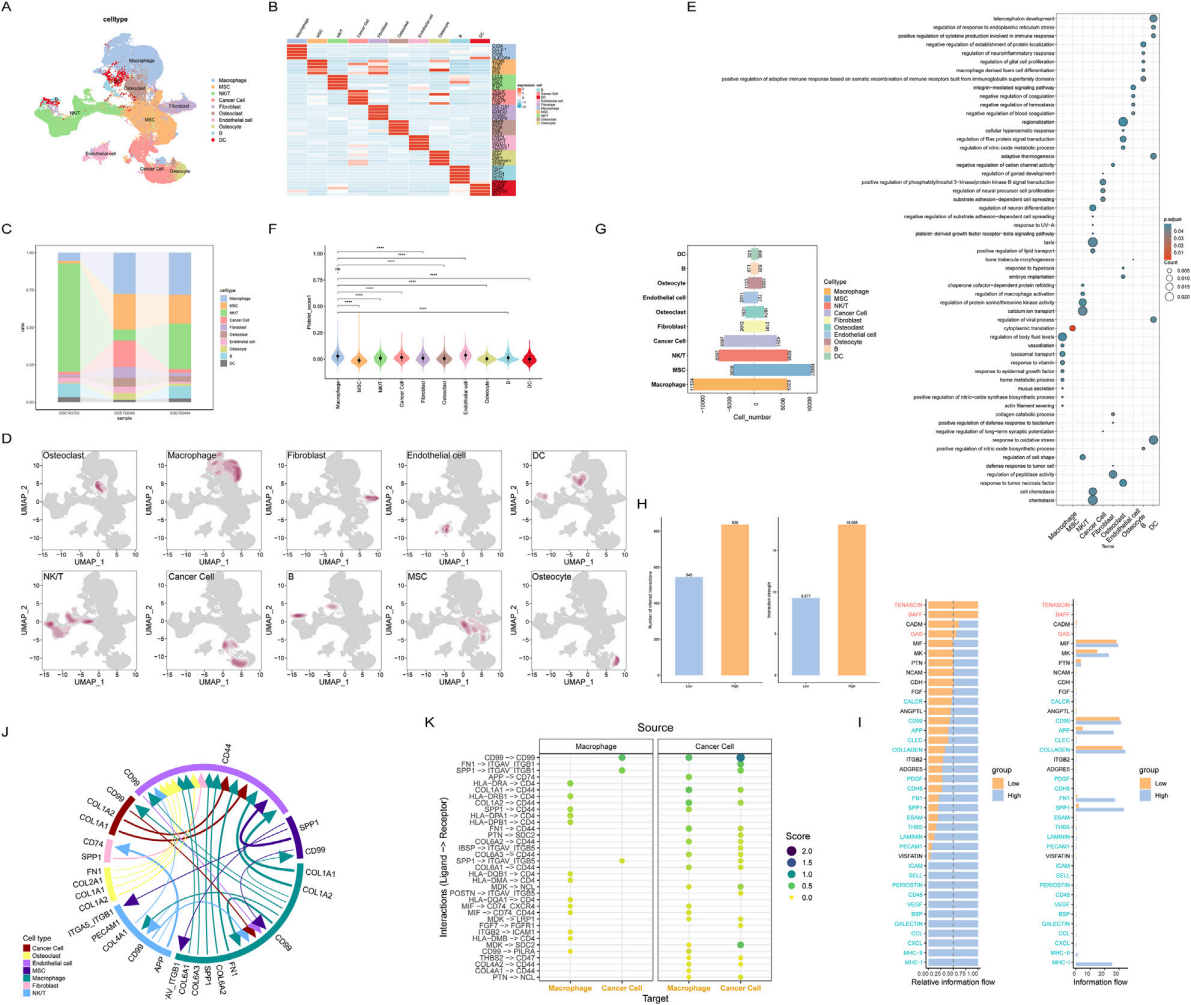
Cell communication between cells with different platelet scores at single-cell resolution; **(A)** Annotated single-cell transcriptomic data; **(B)** Marker genes for each cell subset; **(C)** Distribution of cell types across different datasets; **(D)** Distribution density of each cell subset; **(E)** Enriched pathways for each cell subset; **(F)** Grouping of all cells by platelet score; **(G)** Cells are categorized based on their platelet scores; **(H)** Comparison of communication intensity between cells in different platelet score groups; **(I)** Cell-cell communication pathways between the two groups of cells; **(J)** Major intercellular communication pathways among all cells; **(K)** Intercellular communication between macrophages and tumor cells with high platelet scores.

Ultimately, we obtained the largest osteosarcoma single-cell dataset to date, containing 70,650 cells (Figures 3A, B). The differences in cell types among the three datasets were small, indicating that our data quality control measures were effective (Figure 3C; Supplementary Figure S1E). To explore the biological functions of each cell type, we conducted enrichment analysis on the marker genes of each cell type (Supplementary Figure S2A). The analysis results showed that different cell types had specific enrichments of signaling pathways, indicating significant differences in the transcriptional profiles of these cell types (Figures 3D, E). By using the Addmodulescore function to score platelet activity in each cell, we found that macrophages had the highest scores, significantly higher than all other cell types (Figure 3F).

Each cell was scored using the HALLMARKER50 gene set, and the results showed that the MYC, oxidative stress, and angiogenesis pathways had higher scores in most cell types, suggesting that these cell types may have been reprogrammed in the tumor microenvironment (Supplementary Figure S2B). To investigate the impact of macrophages on the prognosis of osteosarcoma patients, we used the GSVA method to select the top 50 genes of macrophages to calculate the macrophage activity score for each osteosarcoma patient. Cells were divided into two groups, high-scoring and low-scoring, based on the median score of all cells. Due to the small score differences between cells, most cell types were approximately evenly divided into two groups (Figure 3G). After calculating the scores, we used the CellChat package to analyze cell communication between the two groups of cells. The results showed that cell communication was more active in the high-scoring group (Figure 3H), especially the FN1, SPP1, and MHC-I pathways, which were almost exclusively expressed in the high-scoring group, while there was no specific communication in the low-scoring group. This suggests that the high-scoring tumor microenvironment may trigger abnormal cell communication (Figure 3I).

Since macrophages had the highest platelet score, we next focused on exploring the abnormal signaling pathways and cell communication of macrophages. We selected macrophages and tumor cells for analysis, and the results showed that there were many intercellular communications between macrophages and tumor cells, including (Figure 3K).

Finally, to fully understand the intercellular communication between all cell types, we analyzed the most significant intercellular communications between all cell types (Figure 3J). Interestingly, the previously mentioned FN1 and SPP1 appeared in macrophages, MSCs, fibroblasts, and epithelial cells, further validating the abnormal cell communication patterns in macrophages. Additionally, using the top 50 differentially expressed marker genes in macrophages to score the macrophage characteristics for each osteosarcoma patient, the results showed that the higher the macrophage characteristic score, the worse the patient’s prognosis (Supplementary Figure S2C). These results suggest that platelet-related genes are significantly enriched in macrophages and are likely to work synergistically to promote the progression of osteosarcoma.

### TBXAS1 is a key gene affecting the prognosis of osteosarcoma

**FIGURE 4:**
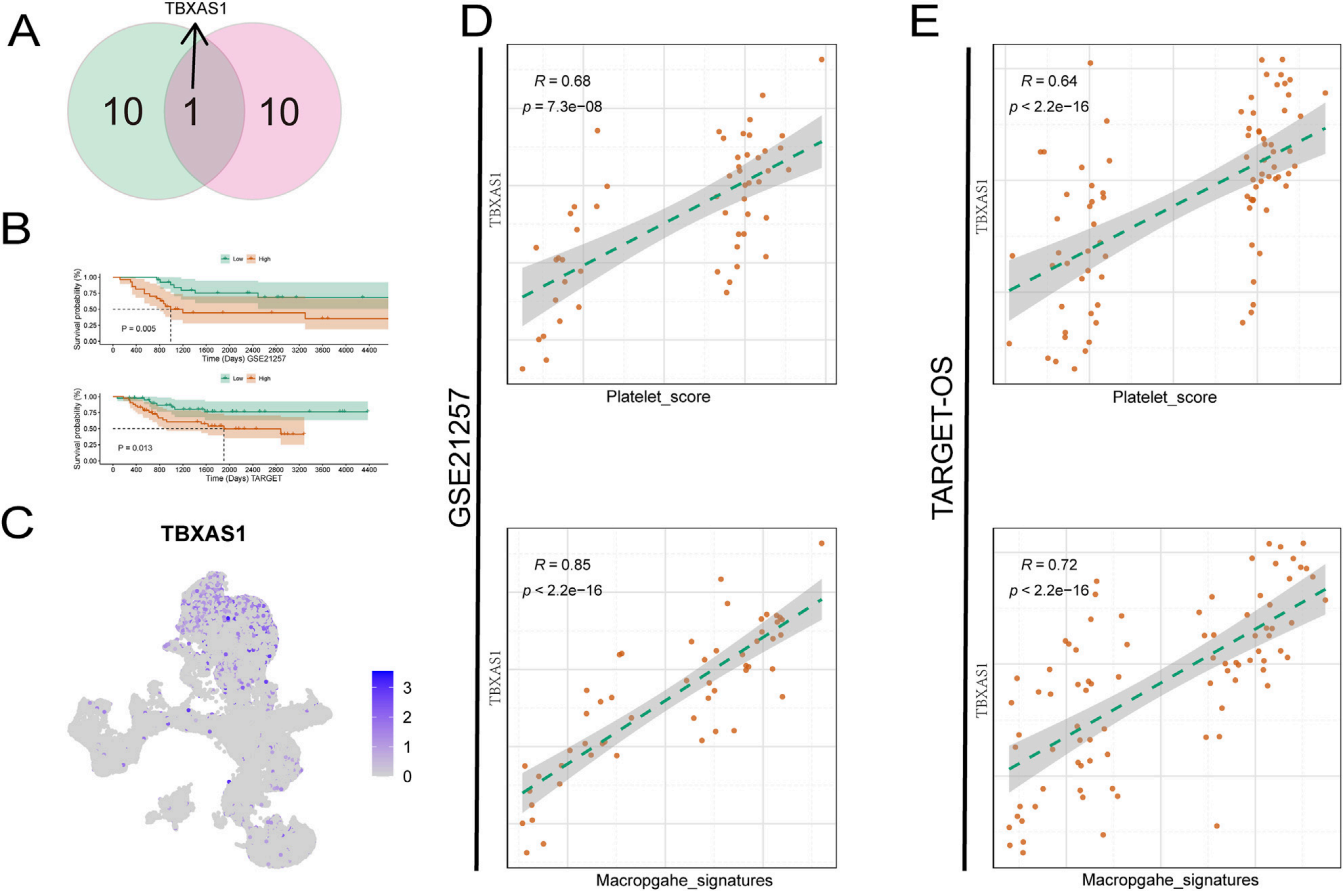
TBXAS1 is a key gene affecting the prognosis of osteosarcoma. **(A)** TBXAS1 is a prognostic gene common to both datasets; **(B)** Survival analysis of different TBXAS1 expressions; **(C)** Distribution of TBXAS1 in low-dimensional single cells; **(D, E)** Correlation analysis between TBXAS1 and macrophage marker gene scores and platelet scores in the two datasets.

Identifying key pathogenic genes, especially in tumors, is a critical research direction for targeted cancer therapy. Therefore, we next analyzed the key genes related to the prognosis of osteosarcoma. When using LASSO regression to determine the weights of various prognosis-related genes, the two datasets obtained different results, which may be due to tumor heterogeneity. We took the intersection of the prognosis genes from the two datasets and found a gene, TBXAS1, that was common to both datasets (Figure 4A). The K-M curve showed that in both datasets, the TBXAS1 gene consistently promoted poor prognosis in patients (Figure 4B).

Next, we used single-cell data to determine the main cell type expressing the TBXAS1 gene, and the results showed that the TBXAS1 gene was mainly expressed in macrophages (Figure 4C). Combining this with the previously mentioned promotional effect of macrophages on osteosarcoma, we propose that TBXAS1 has a potential interaction with macrophages, and this interaction is related to the poor prognosis of osteosarcoma patients. We verified the above hypothesis in the TARGET-OS dataset and the GSE21257 dataset, and the results showed that the expression of TBXAS1 was significantly positively correlated with the marker genes of macrophages (Figure 4D).

**FIGURE 5:**
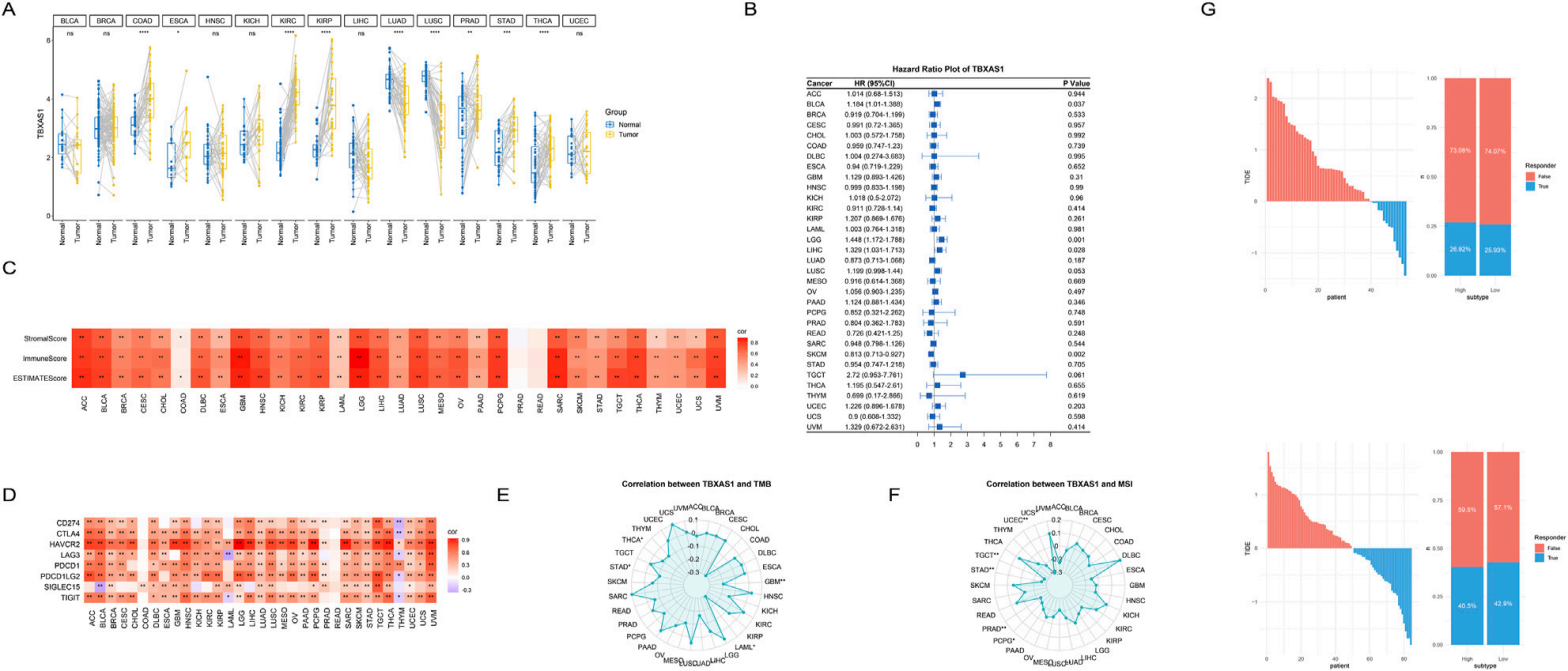
Pan-cancer analysis of the TBXAS1 gene. **(A)** TBXAS1 is significantly differentially highly expressed in various tumors; **(B)** COX regression analysis of TBXAS1 in various tumors; **(C)** Heatmap of the correlation between immune scores of various tumors and TBXAS1 expression; **(D)** Heatmap of the correlation between the expression of immune checkpoints in various tumors and TBXAS1 expression; **(E)** Heatmap of the correlation between TMB (Tumor Mutational Burden) in various tumors and TBXAS1 expression; **(F)** Heatmap of the correlation between MSI (Microsatellite Instability) in various tumors and TBXAS1 expression; **(G)** Relationship between platelet score obtained from TIDE website analysis and immunotherapy response; Top: GSE21257 dataset; Bottom: TARGET-OS dataset.

In addition, there was also a significant positive correlation between platelet score and TBXAS1 expression (Figure 4E). The consistent results from the two datasets support the above hypothesis and are supported by transcriptomic data. Many tumors share key therapeutic targets, such as PD-1/PDL-1, which is widely used. To further explore, we used the transcriptome data of 33 tumors from TCGA database. First, we found that TBXAS1 gene was significantly differentially expressed in nine tumors, of which seven tumors showed significantly increased TBXAS1 expression in tumor tissues, and the other two tumors showed decreased expression (Figure 5A). This suggests that TBXAS1 may play a role in promoting tumor development in most cancers.

To explore the effect of TBXAS1 on the prognosis of each tumor, COX regression analysis was performed for each tumor. The results showed that TBXAS1 was significantly associated with patient prognosis in bladder cancer, low-grade glioma, hepatocellular carcinoma and squamous cell carcinoma. TBXAS1 consistently promoted poor prognosis in all tumors except squamous cell carcinoma, which was consistent with the previous results that TBXAS1 was highly expressed in most tumor tissues (Figure 5B). In addition, with the rapid development of immunotherapy and the increase of successful cases using immune checkpoints to treat tumors, immunotherapy has shown great potential.

Therefore, we used the immune score, matrix score, and estimate score from the estimate package to assess the immune infiltration status of the patients. The results showed that, except for esophageal cancer and pancreatic cancer, the remaining 29 tumors showed significant positive correlations between TBXAS1 and immune scores, stromal scores and tumor purity scores, indicating that TBXAS1 may have a correlation with the immune microenvironment in various tumors (Figure 5C). In addition, the correlation analysis between TBXAS1 and immune checkpoints such as CD274, CTLA4, HAVCR2, LAG3, PDCD1, PDCD1LG2, SIGLEC15 and TIGIT showed that, in addition to thymic cancer, bladder cancer and acute myeloid leukemia, the other 30 tumors had a significant increase in the number of immune checkpoints. TBXAS1 showed a significant positive correlation with the above immune checkpoints (Figure 5D).

The immune checkpoint and estimate score results together illustrate the potential promise of TBXAS1 for immunotherapy. Both TMB (tumor mutation burden) and MSI (microsatellite instability) are important indicators to evaluate tumor characteristics. High TMB usually indicates that the tumor has more genetic mutations, which may affect the recognition of the tumor by the immune system and thus the efficacy of immunotherapy. MSI reflects defects in DNA repair in tumor cells, and MSI-high tumors generally respond better to certain immune checkpoint inhibitors. Evaluation of both can help determine the most appropriate treatment.

Interestingly, in all tumors significantly correlated with TBXAS1 expression, TMB and MSI were significantly negatively correlated with TBXAS1. This result suggests that TBXAS1 gene expression or mutation status has a more direct effect on immune checkpoint regulation rather than an indirect effect on immune responses through mutation burden or microsatellite instability (Figures 5E, F). To verify the effect of TBXAS1 gene on immunotherapy, we entered the gene expression matrix data into TIDE website to calculate TIDE score, and the results showed that more patients with osteosarcoma with high TBXAS1 expression had a response to immunotherapy than those with low TBXAS1 expression (Figure 5G).

## Discussion

The impact of platelets on osteosarcoma is diverse and complex. For example, osteosarcoma cells typically exhibit high platelet activation-induced characteristics, which can induce platelet activation. Activated platelets secrete LPA and CLEC, which enhance the invasive ability of osteosarcoma through the LPA-LPAR1 axis and the interaction between platelet CLEC-2 and osteosarcoma (Takagi et al., 2021). When platelets come into contact with osteosarcoma cells, they secrete various growth factors, such as TGF-β and VEGF, which can induce osteosarcoma cells to express tissue factor and promote tumor growth (Saito et al., 2018). This article integrates single-cell and bulk RNA data to validate the promotional effect of platelets on osteosarcoma and discovers that the possible interaction between the platelet-related gene TBXAS1 and macrophages is a potential mechanism of platelet pro-tumor effects.

Platelets can coordinate the response of monocytes/macrophages to pathological conditions (Carestia et al., 2019; Rossaint et al., 2021). Compared to the circulating monocytes of wild-type mice, the levels of inflammatory factors in circulating monocytes are higher in platelet-deficient mice. Additionally, alveolar platelets are essential for the transcriptional reprogramming and polarization of alveolar macrophages, a process that can lead to the effective resolution of neutrophil clearance and lung inflammation (Rossaint et al., 2021). Currently, there have been several reports on the impact of interactions between platelets and macrophages in the tumor niche. For example, TAMs, which are primarily derived from circulating monocytes, are one of the most abundant immune cells in the CRC microenvironment (Zhao et al., 2021). In the prostate cancer model, platelet-derived growth factor (PDGF) activates TAMs and polarizes them into the M2 phenotype, thereby inducing the growth of prostate cancer cells (Cui et al., 2022). The conclusions drawn in this article are consistent with previous studies, further proving that the interaction between platelets and macrophages is an indispensable process in tumor development.

TBXAS1, also known as thromboxane A synthase 1, encodes a member of the cytochrome P450 enzyme superfamily, which plays an important role in drug metabolism and the synthesis of cholesterol, steroids, and other lipids (Yokoyama et al., 1993). TBXAS1 protein has been reported to be strongly associated with pathophysiological processes, including hemostasis, cardiovascular disease, and stroke. Additionally, it has been reported that TBXAS1 protein is significantly enriched in TAM exosomes, and its expression is related to tumor progression (Chen Q. et al., 2021). In prostate cancer, TBXAS1 expression is associated with the severity of prostate cancer lesions, with the highest levels in advanced and poorly differentiated forms (Dassesse et al., 2006). The enzyme is involved in the migration of prostate cancer cells but not in proliferation or survival (Dassesse et al., 2006). In breast cancer, there is controversy about the correlation between TBXAS1 expression and tumor grading, with one study showing that expression is lost with increasing grade, but there are also opposite reports (Watkins et al., 2005; Li et al., 2017). However, TBXAS1 polymorphisms are moderately associated with breast cancer risk and poor outcomes (Li et al., 2017). The conclusions of the above studies are consistent with the pan-cancer analysis conclusions of this article, indicating that the specific role of TBXAS1 in different tumors is heterogeneous.

In summary, this article used the largest osteosarcoma single-cell data to date to discover that TBXAS1 is a key platelet-related gene leading to poor prognosis in osteosarcoma, filling the gap in the potential role of platelet activity genes in osteosarcoma. Patients with elevated TBXAS1 expression exhibit a significantly higher risk of recurrence and metastasis, underscoring the need for intensified clinical monitoring for this subgroup. Such monitoring programs could enhance the early detection of postoperative recurrence or metastasis, enabling timely intervention and potentially improving overall patient outcomes. Moreover, the functional properties of TBXAS1 position it as a promising therapeutic target for future osteosarcoma treatments. Consequently, this study advocates for further experimental investigations to elucidate the mechanistic role of TBXAS1 in osteosarcoma progression and to explore its feasibility as a novel therapeutic target.

However, this article also has certain limitations. Our results are all based on public databases, and although they have been verified by multiple independent datasets, the stability of their conclusions remains to be discussed. Secondly, we only explored the interaction between macrophages and platelet-related genes, lacking a more in-depth analysis of macrophage subtypes (such as M1 and M2). Additionally, the data used in this article is integrated from multiple datasets, and although batch effects have been removed, the analysis results are inevitably affected.

## Conclusion

This study identified TBXAS1 as a key platelet-related gene responsible for the poor prognosis of osteosarcoma through bioinformatics methods. This gene may affect the prognosis of osteosarcoma patients by interacting with macrophages, providing new insights into the pathogenesis and targeted therapy of osteosarcoma in the future.

## Data Availability

Publicly available datasets were analyzed in this study. This data can be found here: https://www.ncbi.nlm.nih.gov/geo/.
