# Supplementary figures and images for: Integration of single-cell and bulk analysis reveals TBXAS1 as a key platelet-related gene causing poor prognosis in osteosarcoma
Source: Front Genet. 2024 Dec 9;15:1519529. doi: 10.3389/fgene.2024.1519529 (PMC11667113; doi:10.3389/fgene.2024.1519529)

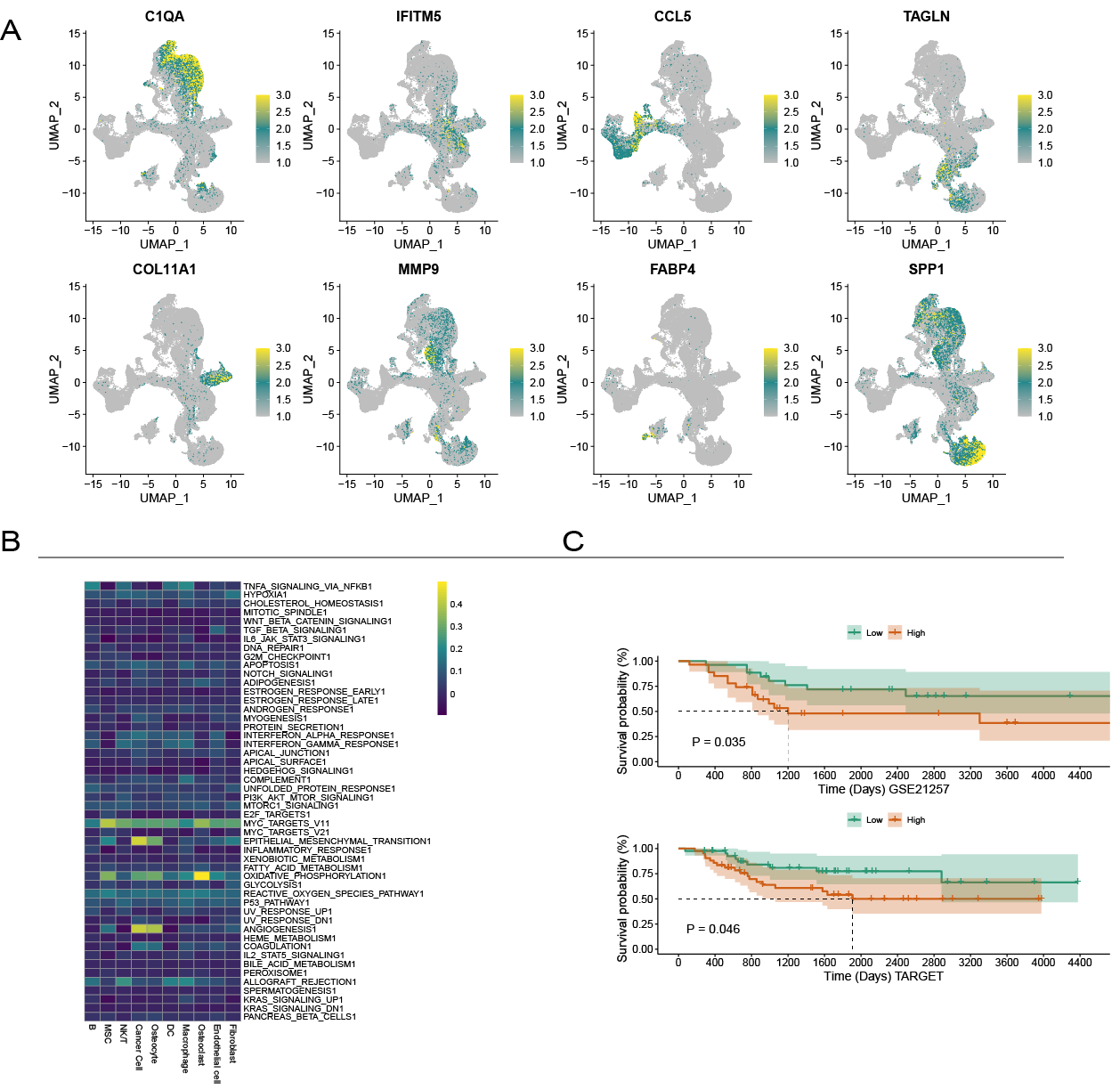

Supplement: Supplementary file 1 [file Image2.tif]
